# Exercise as potential countermeasure for the effects of 70 days of bed rest on cognitive and sensorimotor performance

**DOI:** 10.3389/fnsys.2015.00121

**Published:** 2015-09-03

**Authors:** Vincent Koppelmans, Ajitkumar P. Mulavara, Peng Yuan, Kaitlin E. Cassady, Katherine A. Cooke, Scott J. Wood, Patricia A. Reuter-Lorenz, Yiri E. De Dios, Vahagn Stepanyan, Darcy L. Szecsy, Nichole E. Gadd, Igor Kofman, Jessica M. Scott, Meghan E. Downs, Jacob J. Bloomberg, Lori Ploutz-Snyder, Rachael D. Seidler

**Affiliations:** ^1^School of Kinesiology, University of MichiganAnn Arbor, MI, USA; ^2^NASA Johnson Space CenterHouston, TX, USA; ^3^Universities Space Research AssociationHouston, TX, USA; ^4^Department of Psychology, University of MichiganAnn Arbor, MI, USA; ^5^Department of Psychology, Azusa Pacific UniversityAzusa, CA, USA; ^6^Neuroscience Program, University of MichiganAnn Arbor, MI, USA; ^7^Wyle Science, Technology and Engineering GroupHouston, TX, USA; ^8^Bastion TechnologiesHouston, TX, USA; ^9^Department of Health and Human Performance, University of HoustonHouston, TX, USA; ^10^Institute of Gerontology, University of MichiganAnn Arbor, MI, USA

**Keywords:** cognition, sensorimotor functioning, exercise, bed rest, microgravity, spaceflight analog, longitudinal

## Abstract

**Background**: Spaceflight has been associated with changes in gait and balance; it is unclear whether it affects cognition. Head down tilt bed rest (HDBR) is a microgravity analog that mimics cephalad fluid shifts and body unloading. In consideration of astronaut’s health and mission success, we investigated the effects of HDBR on cognition and sensorimotor function. Furthermore, we investigated if exercise mitigates any cognitive and sensorimotor sequelae of spaceflight.

**Method**: We conducted a 70-day six-degree HDBR study in 10 male subjects who were randomly assigned to a HDBR supine exercise or a HDBR control group. Cognitive measures (i.e., processing speed, manual dexterity, psychomotor speed, visual dependency, and 2D and 3D mental rotation) and sensorimotor performance (functional mobility (FMT) and balance performance) were collected at 12 and 8 days pre-HDBR, at 7, 50, and 70 days in HDBR, and at 8 and 12 days post-HDBR. Exercise comprised resistance training, and continuous and high-intensity interval aerobic exercise. We also repeatedly assessed an outside-of-bed rest control group to examine metric stability.

**Results**: Small practice effects were observed in the control group for some tasks; these were taken into account when analyzing effects of HDBR. No significant effects of HDBR on cognition were observed, although visual dependency during HDBR remained stable in HDBR controls whereas it decreased in HDBR exercise subjects. Furthermore, HDBR was associated with loss of FMT and standing balance performance, which were almost fully recovered 12 days post-HDBR. Aerobic and resistance exercise partially mitigated the effects of HDBR on FMT and accelerated the recovery time course post-HDBR.

**Discussion**: HDBR did not significantly affect cognitive performance but did adversely affect FMT and standing balance performance. Exercise had some protective effects on the deterioration and recovery of FMT.

## Introduction

### Spaceflight Effects on Sensorimotor Performance and Cognitive Function

Spaceflight has been associated with deterioration of locomotor function (Mulavara et al., 2010) and postural stability (Cohen et al., 2012) in astronauts after their return to Earth. These adverse effects of spaceflight have been ascribed to several factors including muscle unloading and reinterpretation of vestibular inputs (Young et al., 1984). Whether spaceflight also induces cognitive deterioration is undecided, but not unlikely, considering the spaceflight-related risks for impaired cognitive function such as chronic stress, sleep deprivation, fluid shifts, and hormone imbalances (Strangman et al., 2014). In addition, spaceflight related neuroplasticity that has been observed in rodents could affect cognitive performance (Holstein et al., 1999). However, a recent review of 32 studies investigating the adverse neurocognitive effects of short term and long term spaceflight concluded that there is no strong support for or against spaceflight-induced cognitive deficits (Strangman et al., 2014). The authors suggested that the lack of strong results could be related to lack of power due to the small sample sizes of these spaceflight studies [median number of astronauts and cosmonauts in the 32 studies was 3 (range: 1–13)]. Considering the health of crewmembers and mission success, especially now that expeditions to Mars are being planned (Parihar et al., 2015), it is important to determine the potential neurocognitive sequelae of spaceflight. We are currently conducting a study investigating the extent, longevity, and neural bases of spaceflight effects on neurocognitive and sensorimotor performance in a group of astronauts and in a terrestrial study using head down-tilt bed rest (HDBR) as a microgravity analog. Here we describe results of the HDBR analog study of which the protocol has been published previously (Koppelmans et al., 2013).

### Bed Rest Effects on Sensorimotor Performance and Cognitive Function

Microgravity analog studies that can be conducted on Earth, such as in HDBR, provide the possibility to estimate the effects of microgravity on cognitive and sensorimotor functioning in relatively large numbers of participants (i.e., relative to the number of available astronauts). HDBR resembles certain characteristics of the microgravity environment in space, such as cephalad fluid shift, cardiovascular deconditioning, and body unloading. Bed rest in the six-degree head-down tilt position is considered the best Earth model to simulate the effects of prolonged microgravity on the human body (Hutchison, 2001). HDBR has been associated with decreased postural stability (Muir et al., 2011; Mulder et al., 2014), increased fall incidence (Mulder et al., 2014), and reduced functional mobility (FMT; Reschke et al., 2009).

The majority of HDBR studies that focused on cognitive outcomes have mainly investigated the effects of 2 weeks of bed rest or less (Lipnicki and Gunga, 2009). The few studies that have investigated longer duration bed rest (here defined as 4 weeks or more) showed both negative and positive effects of bed rest on cognitive performance. Detrimental effects of bed rest have been observed in various cognitive domains, including simple reaction time, mental arithmetic, short term memory, and executive functioning (Lipnicki and Gunga, 2009). Differences in outcomes between HDBR studies can be partly ascribed to differences in study protocols. Shorter duration HDBR studies may not be able to detect some effects that only emerge when the body is the supine position for longer periods of time. In addition, repetitive testing could lead to practice effects which may mask the detrimental effects of HDBR. The mechanisms behind bed rest related cognitive deterioration are not extensively studied. Lipnicki et al. (2009a) suggested that the adverse effects of HDBR on executive functioning that they observed could be related to changes in the frontal cortex. However, despite some studies reporting negative effects of HDBR on cognition, the current literature does not show strong evidence for bed rest induced cognitive deterioration. Conversely, studies that applied relatively long-duration bed rest often showed improvement in cognitive performance which could reflect practice effects resulting from accumulative task exposure (Calamia et al., 2012) or cognitive training that was given before bed rest in some bed rest studies (Lipnicki and Gunga, 2009). Inclusion of normative control subjects who do not participate in bed rest and who are assessed at similar time intervals as HDBR subjects can help distinguishing practice effects form HDBR effects.

### Exercise as Potential Countermeasure for the Effects of Microgravity on Sensorimotor Performance and Cognitive Function

Previous research showed that 25 min of daily locomotion-like activities (heel raises, squats, and hopping exercises in the upright position) during a 5-day HDBR intervention prevented deterioration of postural stability and decreased fall incidence (Mulder et al., 2014). Moreover, simply standing upright for 25 min per day had the same effect as the locomotion-like activities. In a longer duration 30-day HDBR study, DeRoshia and Greenleaf (1993) compared subjects performing aerobic exercise and subjects performing strength exercise to control subjects. They observed improvement in hand and finger tapping performance from pre-HDBR to in-HDBR in all three groups. In addition, improvement in all cognitive domains was reported (i.e., verbal reasoning, encoding visual-spatial ability, pattern comparison, pursuit tracking, and short-term memory) over the course of HDBR (DeRoshia and Greenleaf, 1993). Except for relatively larger improvements in short term memory for both exercise groups, and relatively larger improvements in verbal reasoning and encoding for the control group, there were no differences in cognitive changes over time between the three groups.

Both the studies by Mulder et al. (2014) and DeRoshia and Greenleaf (1993) looked at relatively short duration HDBR interventions. Future planned space missions such as to Mars (Parihar et al., 2015) are of long-duration and it is therefore important to investigate if long duration HDBR is associated with cognitive and sensorimotor problems and if aerobic and strength training mitigate these effects. Aerobic exercise is a candidate countermeasure for the potential effects of long-duration HDBR on sensorimotor performance and cognition, given the positive effects of locomotion on posture instability and fall incidence caused by short-duration HDBR (Mulder et al., 2014) and because exercise intervention studies have reported improved cognitive functioning in various populations, including healthy older adults and those with neurodegenerative diseases (Bherer et al., 2013). We have therefore conducted a study of long duration (i.e., 70 days) HDBR in which we compared five HDBR exercise subjects, five HDBR non-exercise subjects, and nine normative control subjects who do not participate in HDBR (Koppelmans et al., 2013). We aim to determine effects of long-duration HDBR on cognition and sensorimotor performance, and to verify if exercise mitigates any of these potential effects of HDBR. Furthermore, the normative control subjects will allow us to interpret effects of HDBR in consideration of practice effects. Finally, the potential effects of HDBR on neurocognitive performance could reflect both immediate effects of supine orientation as well as potential bio-physiological processes that affect the brain, such as increased intracranial pressure that may need a longer time to manifest (Caprihan et al., 1999). To distinguish the immediate effects of orientation from other effects of HDBR on neurocognitive performance we assessed the normative subjects in the supine as well as the seated position.

## Materials and Methods

### Participants

The HDBR group consisted of ten right-handed males aged 32.9 ± 4.8 years at time of admission (range: 27.6–39.8 years). HDBR control participants were admitted 13 days before starting 70-day, six degrees-HDBR at the NASA bed rest facility, at the University of Texas Medical Branch, Galveston, TX, USA. HDBR exercise subjects (see below under “exercise intervention”) were admitted 21 days pre-HDBR. All HDBR subjects were dismissed 14 days after HDBR. While in bed rest, participants remained in the head down-tilt position at all times except for 30 min at each meal (three times per day), when they were permitted to support their head with their hands. These individuals participated in multiple experiments while in bed rest, organized by the NASA Flight Analogs Project team. All subjects received monetary compensation for their participation.

Nine male subjects participated as normative control participants (subsequently referred to as the “normative control group”). Their average age was 39.1 ± 8.7 years at time of admission (range: 26.2–55.9 years). The normative participants were recruited from the NASA Johnson Space Center subject test facility.

All bed rest and normative subjects passed an Air Force Class III equivalent physical examination. Both the bed rest study and the normative study were conducted in accordance with the declaration of Helsinki, and were both approved by the institutional review boards of the University of Michigan, the University of Texas—Medical Branch (UTMB), and NASA. Written informed consent was obtained from all participants.

### Exercise Intervention

Bed rest subjects were randomly assigned to either a HDBR exercise group (subsequently referred to as the “exercise group”) or a HDBR control group (subsequently referred to as the “HDBR control group”). Participants in the exercise group started supine exercising 20 days before HDBR. Supine exercise refers to exercise during which the body remained in supine position. The intensity of exercise gradually increased during these 20 days, and full exercise began with the start of HDBR. A detailed description of our exercise program has been described previously (Ploutz-Snyder et al., 2014). In short: during HDBR, exercise participants exercised 6 days per week. On days 1, 3 and 5, resistance training and continuous aerobic exercise were performed, which were separated by at least 4 h. Resistance exercise lasted 35–60 min per training day, including supine squat, heel raise, leg press, and hamstring curl. The intensities of the aerobic exercise were prescribed as heart rate at a percentage of peak oxygen consumption (VO_2peak_) that was measured pre-HDBR during an upright cycle peak-test. Each session of continuous aerobic training was 30 min, at least 75% of VO_2peak_. On days 2, 4 and 6, high-intensity interval aerobic exercise was performed using a vertical treadmill and a supine cycle ergometer, with interval durations of 2 min (six times, at 70, 80, 90, 100, 90 and 80% of VO_2peak_ with a 2 min rest), 30 s (eight times at maximal effort with 15 s of active rest), and 4 min (four times at a target intensity of 85% VO_2peak_), respectively. The test operators adjusted treadmill speed and cycle ergometer load during the exercise session to meet the target heart rate of the continuous and interval sessions. The duration of active rest (i.e., activity at ~40% of VO_2peak_) between two exercise intervals was 2 min, 15 s, or 3 min, respectively, resulting in total exercise sessions of 32 min, 15 min and 35 min on days 2, 4, and 6. No training was performed on day 7. Normative control subjects were not participating in an exercise intervention, but might have exercised as per their own daily routines (data not recorded).

### Cognitive and Sensorimotor Tests

**Figure 1:**
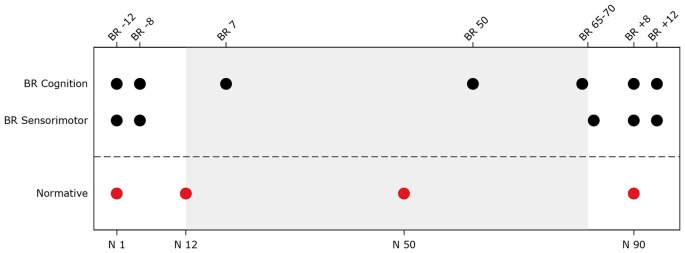
**Testing timeline for head down tilt bed rest (HDBR) and normative subjects**. The top x-axis shows time in days for the bed rest subjects (e.g., BR -8 = 8 days pre-HDBR). The bottom x-axis shows time in days for normative subjects. The gray background indicates the bed rest period. BR Cognition = Time points at which cognitive assessments (i.e., digit symbol substitution test, Purdue pegboard test, rod and frame test, cube rotation test, and card rotation test) took place for the bed rest subjects; BR Sensorimotor = Time points at which sensorimotor tests (i.e., functional mobility test, and sensory organization test (SOT-5 and SOT-5M)) took place for the bed rest subjects; Normative = Time points during which normative subjects completed cognitive tests in supine and seated position and sensorimotor tests.

**Figure 2:**
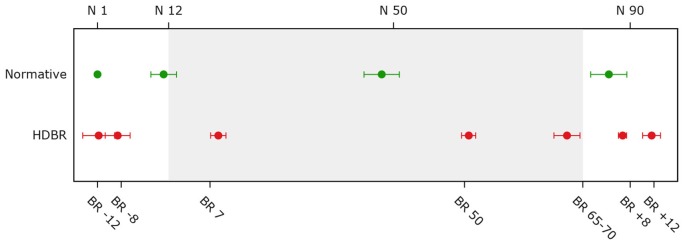
**Average assessment days for bed rest and normative control subjects**. The top x-axis shows time in days for normative subjects. The bottom x-axis shows time in days for the bed rest subjects (e.g., BR -8 = 8 days pre-HDBR). The gray background indicates the bed rest period. Error bars represent standard deviations; Normative = normative control subjects (*n* = 9); HDBR = head down bed rest subjects (*n* = 10).

For the bed rest participants, several behavioral tests were repeatedly administered at two sessions pre-, three sessions during, and two sessions post-bed rest. Normative control subjects were tested at four time points. The testing time lines are presented in Figure 1. Subjects were assessed as close as possible to the planned assessment day. Nevertheless, there was some variation (see Figure 2). Within normative control subjects we evaluated the influence of subject position (supine vs. seated) on behavioral measures to identify to what extent the adverse effects of HDBR can be attributed to the direct result of an acute change in subject position or the effect of accumulative time in HDBR. Cognitive tests for which data were collected in both supine and seated position were administered on two consecutive days. The order of subject position was counterbalanced across participants. The behavioral measures have been described previously (Koppelmans et al., 2013). Participants performed the following tests;

#### Digit Symbol Substitution Test

The Digit symbol substitution test (Lezak et al., 2004) was used to assess processing speed. Participants were provided a key of nine digit-symbol pairs, followed by a list of 140 digits. The paper and pencil were presented in front of the subject while he was in the supine position. The material was mounted on a board that could be height-adjusted for each subject. Subjects were asked to write down the corresponding symbols for these digits as fast as possible. The completion time and number of correct answers were indicators of processing speed.

#### Purdue Pegboard Test

The Purdue pegboard test was used to evaluate bimanual coordination (Tiffin and Asher, 1948). The test consists of a pegboard with parallel rows of 25 holes. The board was mounted in front of the subject and could be adjusted in height. Subjects were instructed to place small cylindrical metal pegs into the holes using both hands simultaneously. The board and pegs were presented in front of the subject while he was in the supine position. The time taken to complete 15 pairs was measured.

#### Rod and Frame Test

The rod and frame test (RFT; Isableu et al., 1998) was used to measure individual reliance on visual vs. other cues (vestibular, proprioceptive). The test consists of a rotatable rod inside a square frame, which can be rotated independently. Subjects were instructed to observe the rod through the tunnel-like frame, so that the visual cues outside of the frame could be removed. The RFT was repeated eight times with two random starting incline positions of the rod and/or the frame (±18°). Participants were lying on their left side to complete the test and were instructed to align and position the rod to match their perceived upright vertical orientation. Subjects were scored in degrees of deviation from true vertical.

#### 3D Cube Mental Rotation Task

In the cube rotation task, subjects were asked to compare assemblages of 3-dimensional (3D) cubes (Shepard and Metzler, 1988). In each trial, a 3D shape was presented on the computer screen for 3 s, followed by a 2-s blank screen and then two 3D cube images. One of the two images was the same as the initial image however it was rotated in 3D space. Participants were lying on their left side to be able to view the screen and were requested to determine which of the two images matched the target. Response time and accuracy were indicators of performance.

#### Thurstone’s 2D Card Rotation Task

This test measured the spatial working memory of the subjects (Ekstrome et al., 1976). The material was mounted on a board that could be height-adjusted for each subject. In each trial, a two-dimensional (2D) drawing of an irregularly shaped card was presented. To the right there were six drawings of the same cards that were either only rotated, or rotated and mirrored. Subjects were asked to identify which cards matched the initial drawing (i.e., which were rotated in 2D space and not mirrored). The paper and pencil were presented in front of the subject while he was in the supine position. Response time and accuracy were measured as indicators of performance.

#### Functional Mobility Test

The FMT test consists of a series of locomotor challenges (Mulavara et al., 2010). Subjects walked through an obstacle course; the first part was set up on a hard floor, whereas the second part was set up on a base of medium density foam to increase postural challenge. The 6.0 m × 4.0 m course consisted of several foam obstacles such as hurdles, pylons and bars. Participants were instructed to walk through the course as quickly and safely as possible, without touching any of the obstacles. Although subjects performed this test repeatedly three times, we considered the first test time as the metric most sensitive to change.

#### Sensory Organization Test

Balance control was measured using the Sensory Organization Tests (SOTs) provided by EquiTest System platform (NeuroCom, Clackamas, OR, USA; Neurocom, 2012). During testing, subjects were instructed to maintain a stable upright posture for 20-s trials with feet positioned shoulder width apart, eyes closed and arms folded across the chest. All trials were conducted with a sway-referenced support surface that was intended to disrupt somatosensory feedback and therefore reflect how well vestibular input could be utilized to maintain balance. The center of pressure in both anterior-posterior and medial-lateral directions was obtained from the force plate and then filtered to estimate the center of mass (COM). The subject’s sway angle was then derived from the COM that was assumed to be above the support surface at approximately 55% of total height (McGinnis, 2013, p149). The anterior-posterior peak-to-peak sway angle was used to compute a continuous equilibrium score scaled relative to a maximum theoretical peak-to-peak sway of 12.5° and normalized by the percent of the trial completed (Wood et al., 2012). In addition to three trials with head erect (referred to as SOT-5), subjects completed three trials while they were tasked to pitch their heads ±20° at 0.33 Hz as cued by an oscillating tone provided over headphones (referred to as SOT-5M). SOT-5M is more difficult than the SOT-5 by requiring voluntary head movements and requiring integration of both semicircular canal (angular) and otolith (linear) cues. For both the SOT-5 and SOT-5M, we selected the median score of the three trials to prevent effects of outliers.

### Statistical Analyses

Both accuracy and speed outcome measures of the card rotation test and performance on SOT-5M were negatively skewed due to ceiling effects for the card rotation test and near to ceiling effects for SOT-5M. Reverse score transformation with subsequent log or square root transformation did not yield normally distributed data. For lack of an appropriate alternative analysis model we maintained the linear-mixed model (see below). Therefore, results of these outcome measures should be interpreted with caution. Data of all remaining outcome measures were normally distributed.

#### Effects of Group and Time in HDBR Subjects and Normative Subjects

Linear mixed model analysis was used to test group by time differences between HDBR exercise and HDBR control subjects, and to test subject position by time differences for the normative control subjects. The subject variable was entered as a random intercept. For each outcome measure, we tested the simple effects of time, group, and group by time. *Post hoc* pairwise comparisons of time, group, and group by time were only conducted if their respective simple effect was significant at *p* < 0.05. If the interaction was significant we explored the time courses for the HDBR exercise group and HDBR control group separately in addition to the pairwise interaction effects. We selected the second baseline measure (i.e., 8 days pre-bed rest for HDBR subjects and 12-day follow-up for the normative subjects) as the reference time point for the *post hoc* pairwise comparisons to account for practice effects that were expected from the first to the second assessment. To correct for alpha inflation we applied Bonferroni correction.

#### HDBR Subjects vs. Normative Control Subjects

The time intervals between test dates differed for the HDBR and normative control subjects, because the control testing timeline was optimized for another study. Due to this, we used trend analysis in which time was entered as a continuous variable to compare the time courses of both HDBR exercise and HDBR control subjects to the time course of the normative control subjects. For this analysis, we included data from the second measurement onwards for both groups to ensure that all subjects had at least one practice session. In addition, we excluded the post-HDBR time points for this analysis because we were interested in comparing performance over the course of HDBR to normative performance over a similar time course. Because of age differences between HDBR subjects and normative control subjects this analysis was adjusted for age at baseline (i.e., BR -12 for HDBR subjects and normative assessment one for normative control subjects).

Restricted maximum likelihood (REML) was used in all linear mixed model analyses because REML estimation is less sensitive to small sample bias than traditional maximum likelihood due to fixed-effects estimation (Van Dongen et al., 1999). Alpha levels were set at 0.05 for all analyses. Stata was used for all analyses (StataCorp. 2013. Stata Statistical Software: Release 13. College Station, TX, USA: StataCorp LP).

## Results

Table 1 provides demographics of the HDBR groups and the normative control subjects as well as baseline scores for both groups. Table 2 gives an overview of the main effects of group, time, and the interaction effect of group by time for both HDBR groups, and an overview of the main effects of subject position, time, and the interaction effect of subject position by time for the normative control subjects. For the HDBR groups, significant main effects of time were observed for all outcome measures except for the accuracy measure of the card rotation test. Significant effects of group were observed for the Purdue pegboard test, the accuracy measure of the rod and frame test, and SOT-5. Significant interaction effects were observed for the accuracy measure of the rod and frame test, and all FMT outcome measures except for time needed to complete the second half of the FMT. For normative control subjects, main effects of time were observed for all cognitive measures except for the rod and frame test, time needed to complete the second half of the FMT, and SOT-5. Significant main effects of subject position were observed for the Purdue pegboard test and the accuracy measure of the rod and frame test. In addition, a significant interaction was observed for the Purdue pegboard test.

**Table 1:** **Demographics and baseline scores for bed rest subjects and normative control subjects**.

		HDBR subjects				Normative control subjects			
		Control		Exercise		Seated position		Supine position	
		(between subjects design)				(within subjects design)			
**N**		5		5		9			
**Male**	%	100%		100%		100%			
**Age—mean (SD)**	Years	33.7	(5.4)	32.1	(4.5)		39.1	(8.7)	
**Baseline (i.e., 2^nd^ assessment):**		8 days pre HDBR				Normative day 12			
**Cognitive tests**	**Unit**	**Mean**	**(S.E.)**	**Mean**	**(S.E.)**	**Mean**	**(S.E.)**	**Mean**	**(S.E.)**
Digit symbol substitution test	Time (s)	237.21	(18.8)	209.9	(18.8)	180.6	(9.8)	193.6	(9.8)
Purdue pegboard test	Time (s)	60.8	(3.8)	51.4	(3.8)	43.0	(3.7)	61.3	(3.7)
Rod and frame test	Deviation (degrees)	17.5	(1.6)	14.9	(1.6)	4.4	(1.3)	17.9	(1.3)
3D cube rotation test	Time (s)	3.2	(0.5)	2.6	(0.5)	3.3	(0.2)	3.3	(0.2)
3D cube rotation test	% Correct	81.5	(4.9)	83.8	(4.9)	86.8	(3.5)	85.0	(3.5)
2D card rotation test	Time (s)	172.2	(10.8)	169.5	(10.8)	155.3	(8.1)	160.0	(8.1)
2D card rotation test	% Correct	76.6	(7.9)	88.5	(7.9)	96.7	(1.5)	93.7	(1.5)
**Sensotimotor tests**	**Unit**	**Mean**	**(S.E.)**	**Mean**	**(S.E.)**		**Mean**	**(S.E.)**
Functional mobility test total	Time (s)	21.1	(2.2)	21.3	(2.2)		24.7	(1.3)
Functional mobility test—1st half	Time (s)	10.6	(1.1)	11.3	(1.1)		12.6	(0.6)
Functional mobility test—2nd half	Time (s)	10.5	(1.2)	10.0	(1.2)		12.1	(0.8)
Sensory organization test 5	%	76.1	(3.1)	86.6	(3.1)		85.3	(1.8)
Sensory organization test 5—with head movement	%	68.4	(7.1)	82.6	(7.1)		77.5	(3.2)

*HDBR, head down bed rest; SD, standard deviation; S.E., standard error*.

**Table 2:** **Main effects of group, time and group by time interaction for HDBR and normative subjects**.

		DSS	PPB time (s)	RFT deviation (degrees)	Cube time (s)	Cube accuracy	Card time (s)	Card accuracy	FMT total time (s)	FMT 1st half time (s)	FMT 2nd half time (s)	SOT-5 %	SOT-5M %
**HDBR subjects**
Group	χ^2^		4.3	5.5								13.0
df = 1	*p*		0.039	0.019								<0.001
Time	χ^2^	84.4	56.2	19.8	29.4	18.8	88.0		57.6	45.5	54.7	19.4	58.4
df-c = 6; df-s = 4	*p*	<0.001	<0.001	0.003	<0.001	0.005	<0.001		<0.001	<0.001	<0.001	<0.001	<0.001
Group × Time	χ^2^			15.4					13.2	16.5
df-c = 6; df-s = 4	*p*			0.017					0.011	0.002
**Normative subjects**
Position	χ^2^		16.2	169.5			6.5		–	–	–	–	–
df = 1	*p*		<0.001	<0.001			0.011
Time	χ^2^		31.2		22.7	10.5	36.2	8.3	10.2	13.0			8.6
df = 3	*p*		<0.001		<0.001	0.015	<0.001	0.041	0.017	0.005			0.035
Position ×	χ^2^		8.1						–	–	–	–	–
Time df = 3	*p*		0.045

*df, degrees of freedom; df-c, degrees of freedom for cognitive tests; df-s, degrees of freedom for sensorimotor tests; DSS, digit symbol substitution test; PPB, Purdue pegboard test; RFT, rod and frame test; Cube, 3D cube rotation test; Card, 2D card rotation test; FMT, functional mobility test; SOT-5, sensory organization test 5; SOT-5M, sensory organization test 5 with head movement*.

**Figure 3:**
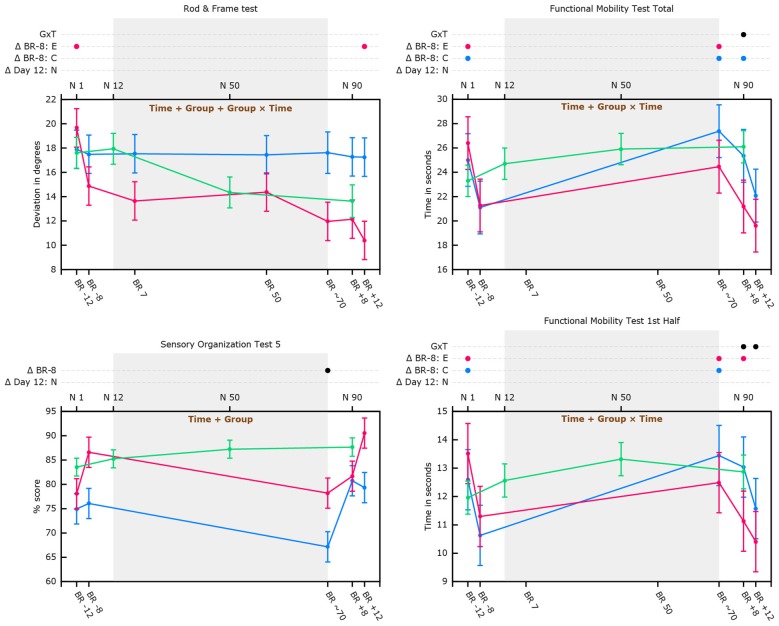
**Cognitive and sensorimotor performance as a function of bed rest and in normative non-bed rest subjects in supine position**. Graphs show marginal means with pooled standard errors. The top x-axis shows time in days for normative subjects. The bottom x-axis shows time in days for the bed rest subjects (e.g., BR -8 = 8 days pre-HDBR). Blue lines represent data of HDBR control subjects; Red lines represent data of HDBR exercise subjects; Green lines represent data of normative control subjects (in supine position for the Rod and Frame graph); Text printed in brown within the graphs show which main effects are significant for the HDBR analyses (e.g., time, time + group, or group + time + group × time). Dots (e.g., ●) indicate at which time point(s) values differ significantly from their baseline (Δ BR -8 = 8 days pre-HDBR for the total group of HDBR subjects; Δ Day 12: N = day 12 for normative control subjects). In case of a significant group by time interaction effect or group effect of exercise separate lines are presented for HDBR control subjects (in blue; Δ BR -8: C) and HDBR exercise subjects (in red; Δ BR -8: E). Δ G×T indicates time points with significant group by time interaction.

Table 3 shows differences from baseline (i.e., 8 days pre-HDBR) in cognitive and sensorimotor performance measures in the HDBR group. Table 4 shows differences from baseline (i.e., Day 12) in cognitive and sensorimotor performance measures in the normative control group. Table 5 shows significant interaction effects of time in days entered as continuous variable, and group (i.e., normative control subjects, HDBR exercise and HDBR control subjects) in our trend analyses for sensorimotor outcome measures. No significant interactions in this trend analysis were observed for cognitive outcome measures. Figure 3 shows a graphical representation of the significant group-by-time interactions presented in Tables 1–4.

**Table 3:** **Interaction effects between bed rest with and without exercise countermeasure on cognitive and sensorimotor performance**.

		DSS	PPB time (s)	Cube accuracy	Card time (s)	FMT total time (s)	FMT 1st total time (s)	FMT 2nd total time (s)	SOT-5 %	SOT-5M %
**Group:^a^**
Δ Exercise	β								10.53
	*p*								0.016
**Time:^b^**
Δ BR^−12^	β	38.39		−13.85		3.91	2.00	1.94		−20.20
	*p*	0.002		0.007		0.002	0.001	0.011		0.003
Δ BR^7^	β					–	–	–	–	–
	*p*
Δ BR^50^	β					–	–	–	–	–
	*p*
Δ BR^~70^	β		−5.15		−17.61	6.28*	2.82*	3.61*	−8.92	−29.88*
	*p*		0.041		0.042	<0.001	<0.001	<0.001	0.040	<0.001
Δ BR^+8^	β		−5.77		−27.15	4.25	2.41*	1.84
	*p*		−5.77		0.002	4.25	<0.001	0.017
Δ BR^+12^	β	−28.38	−5.87		−26.32
	*p*	0.025	0.020		0.002
**Interaction:^c^**
at BR^+8^	β					−4.33	−2.57
						0.016	0.003
	β					−4.33	−2.57
	*p*					0.016	0.003
at BR^+12^	β						−1.84
	*p*						0.032

^a^Difference compaired to bed rest control subjects; ^b^difference from BR^−8^; ^c^difference from control subjects at BR^−8^; DSS, digit symbol substitution test; PPB, Purdue pegboard test; Cube, 3D cube rotation test; Card, 2D card rotation test; FMT, functional mobility test; SOT-5, sensory organization test 5; SOT-5M, sensory organization test 5 with head movement; BR, bed rest; β, parameter estimate; ^*^, effects that reamined significant after Bonferroni correction for multiple testing; *Note: columns for the rod and frame test, speed of the cube rotation task and accuracy of the card rotation task are not presented because none of the post hoc tests was significant*.

**Table 4:** **Effects of subject position and time on cognitive performance in normative control subjects**.

		PPB time (s)	RFT deviation (degrees)	Cube accuracy	Card time (s)	SOT-5M%
**Group:^a^**
Δ Supine	β	18.23	13.56			−
	*p*	<0.001	<0.001			−
**Time:^b^**
Δ N^1^	β			−9.40		7.40
	*p*			0.006		0.039
Δ N^50^	β
	*p*
Δ N^90^	β				−10.34
	*p*				0.033

*^a^Difference compaired to seated position; ^b^difference from N^−12^; PPB, Purdue pegboard test; RFT, rod and frame test; Cube, 3D cube rotation test; Card, 2D card rotation test; SOT-5M, sensory organization test 5 with head movement; BR, bed rest; β, parameter estimate; Note: no post hoc tests for interaction were significant; columns for the digit symbol substitution test, speed of the cube rotation task, accuracy of the card rotation task, the functional mobility test, and the senosry organization test 5 without head movement are not presented because none of the post hoc tests was significant*.

**Table 5:** **Significant interaction effects resulting from trend analysis of sensorimotor tests**.

	Group	β Group × Time	S.E.	*p*
FMT—Total	BR Control	0.07	0.03	0.017
Time (s)	BR Exercise	0.02	0.03	n.s.
FMT—1^st^ Half	BR Control	0.04	0.01	0.012
Time (s)	BR Exercise	0.01	0.01	n.s.
FMT—2^nd^ Half	BR Control	0.04	0.02	0.048
Time (s)	BR Exercise	0.01	0.02	n.s.
SOT-5%	BR Control	−0.15	0.06	0.014
	BR Exercise	−0.14	0.06	0.021
SOT-5M%	BR Control	−0.39	0.12	0.001
	BR Exercise	−0.33	0.12	0.004

*Normative control subjects were the reference group. Time, time in days from baseline (BR -8 for HDBR subjects; day 12 for normative control subjects); S.E., standard error of the parameter estimate; FMT, functional mobility test; SOT-5, sensory organization test 5; SOT-5M, sensory organization test 5 with head movement; n.s., not significant; Analyses were adjusted for age*.

### Pre-HDBR Changes

Except for the card rotation test, time needed to complete the cube rotation test and SOT-5, pairwise comparisons revealed that HDBR exercise subjects showed a significant improvement from 12 days pre-HDBR to 8 days pre-HDBR on all outcome measures, suggesting practice effects for these tasks. HDBR control subjects improved significantly from 12 days pre-HDBR to 8 days pre-HDBR on the same outcome measures as the exercise subjects, except for performance on the rod and frame test.

### Digit Symbol Substitution Test

Due to a ceiling effect for this this test we were not able to model accuracy as a function of HDBR over time in the normative subjects. Compared to 8 days pre-HDBR, HDBR subjects completed the task significantly faster at 8 and 12 days post-HDBR, which likely reflects practice effects. Normative control subjects did not show significant improvement after their second assessment (i.e., at day 12) and performance was not associated with subject position.

### Purdue Pegboard Test

Compared to 8 days pre-HDBR the HDBR subjects performed better at the last day in HDBR and at 8 and 12 days post HDBR. Normative control subjects performed significantly better in seated position than in supine position. Performance in seated position did not significantly differ at any time point from the second assessment and only performance at the first day in supine position was significantly worse than performance at day 12. Although there was a significant interaction effect between time and subject position, follow-up analysis did not reveal any particular time point at which this interaction was significant.

### Rod and Frame Test

Although there were significant main effects of group, time, and group by time interaction, follow-up analyses did not reveal any particular time point at which these main effects were significant. Analysis stratified by HDBR group however revealed that whereas rod alignment to actual vertical over the course of HDBR or post-HDBR did not change for HDBR control subjects, exercise subjects became better at aligning the rod to Earth vertical. Post-HDBR, this improvement reached significance (see Figure 3). For normative control subjects there was no significant main effect of time. Overall, supine performance resulted in significantly worse performance.

### 3D Cube Rotation Test

There were no significant differences in accuracy or speed of cube rotation performance between the HDBR exercise and HDBR control group. Time needed to complete the cube rotation test was significantly less at 70 days in HDBR and post-HDBR compared to 8 days pre-HDBR. Cube rotation accuracy was not significantly different from 8 days pre-HDBR at any time during or post-HDBR. Normative subjects’ speed or accuracy did not change from day 12 to assessments at day 50 or 90.

### 2D Card Rotation Test

Time needed to complete the card rotation test did not significantly differ between HDBR exercise and HDBR control subjects. Compared to 8 days pre-HDBR, performance was faster at all consecutive time points, although performance was not associated with time. In the normative control subjects time needed to complete the card rotation test was only significantly less at day 90 compared to day 12. Accuracy was not associated with time.

### Functional Mobility Test

HDBR exercise and HDBR control subjects needed significantly more time to complete the FMT (all outcome measures) directly after 70 days in-HDBR compared to 8 days pre-HDBR (see Figure 3). At 8 days post-HDBR, HDBR control subjects still needed significantly more time than 8 days pre-HDBR, whereas the exercise subjects already had significantly improved their scores for the total FMT at 8 days post-HDBR compared to 8 days pre-HDBR (see Figure 3). At 12 days post-HDBR control subjects had also significantly improved from baseline. None of the FMT outcome measures showed a significant effect of exercise, but significant group by time interactions were observed for time needed to complete the total FMT at 8 days post-HDBR, indicating larger improvement for the exercise group. In addition, group by time interactions were observed for time needed to complete the first half of the FMT at 8 and 12 days post-HDBR, indicating improvements for the exercise group and deterioration for the HDBR control group. Normative control subjects did not show significant improvements from assessment at 12 days onwards (see Figure 3). Trend analysis showed that relative to slope of the change in FMT performance of normative control subjects, HDBR control subjects needed more time to complete these tests post HDBR. This was not the case for HDBR exercise subjects (see Table 5).

### Sensory Organization Tests

We observed a significant effect of group indicating that HDBR exercise subjects’ SOT-5 performance was overall better then performance of HDBR control subjects. Compared to 8 days pre-HDBR, SOT-5 and SOT-5M performance was significantly worse directly post-HDBR (see Figure 3). No significant changes from 8 days pre-HDBR were observed at 8 or 12 days post-HDBR for either SOT-5 or SOT-5M. SOT-5 scores of normative control subjects were not associated with time and their SOT-5M scores did not significantly change from baseline (i.e., day 12) to day 50 or day 90. Trend analysis revealed that relative to normative control subjects, SOT-5 and SOT-5M performance of both HDBR exercise and HDBR control subjects significantly linearly declined from pre-HDBR to immediately post-HDBR (see Table 5).

### Alpha Inflation

After correction for multiple comparisons the effects of HDBR and exercise on cognitive performance were no longer significant. However, the detrimental effects of HDBR on FMT performance (time needed to complete the total, first and second half of the FMT) that were observed immediately post-HDBR and 8 days post-HDBR (time needed to complete the first half of the FMT) as well as deterioration of balance performance with head movement at 8 days post-HDBR remained significant after Bonferroni correction. The observed cognitive and sensorimotor changes over time or those related to position in normative subjects did not survive Bonferroni correction.

## Discussion

We investigated whether exercise in the supine position before and during HDBR mitigates any potential adverse effects of HDBR on sensorimotor performance and cognitive functioning and how this relates to learning effects in normative control subjects who did not participate in HDBR or an exercise intervention. Furthermore, within these normative control subjects we investigated how performance is affected by completing cognitive tests in the supine position compared to completing them in a seated position, in order to explore the subject position-related direct effects of long-term HDBR on performance as opposed to the long(er)-term effects that might be related to physical deconditioning (Convertino et al., 1997) and central nervous system (CNS) changes potentially resulting from prolonged inactivity (Roberts et al., 2010; Rao et al., 2014).

### Cognitive Performance

No significant deterioration in cognitive performance was observed from 8 days pre-HDBR to any time during HDBR. On the digit symbol test, which measures manual dexterity and psychomotor speed, we observed a main effect of exercise. This indicates that exercise subjects were overall faster. It is unclear whether this group difference represents effects of exercise, or differences at baseline, or both. Randomization of subjects should have resulted in similarity of the groups at baseline, although this might have been hampered by our small sample size (Moher et al., 2010; de Boer et al., 2015). It is also important to note that subjects randomized to the exercise condition started ramping up their exercise training immediately upon study admission. It is possible, perhaps even likely, that beneficial effects of exercise had already occurred by 12 and 8 days pre-HDBR as subjects had been gradually increasing their exercise over the 9 days prior to their first tests.

Results from the rod and frame test showed that visual dependency remained stable in HDBR control subjects but declined in HDBR exercise subjects. Previous research showed that subjects who are more visually dependent tend to have greater difficulty adapting to novel discordant sensory stimuli (Brady et al., 2012). Thus, the decrease in visual dependency that we observed in the group of HDBR exercise subjects could reflect a beneficial development in terms of sensorimotor integration and function. Because HDBR control subjects did not become less visually dependent with accumulating time in HDBR, it seems reasonable to assume that exercise mitigates the effects of bed rest on practice effects of visual dependency. However, change in visual dependency in normative subjects was not significant and there were no significant interactions between normative control subjects and HDBR exercise and control subjects. We can therefore only speculate about effects of HDBR on visual dependency and further research is necessary to validate these non-significant finings. Interestingly, the normative control subjects performed worse in supine position than in seated position. These data thus indicate that change of posture can affect visual dependency in such a way that transitioning into the supine position results in being more visually dependent. Increased visual dependency has been observed previously in astronauts post-flight, as an adaptation to diminished gravitational cues (Young et al., 1986). Potentially, the mitigating effect of exercise could help astronauts recover post-flight in stabilizing and orienting posture because they are better able to integrate information from several sensory systems (Isableu et al., 2008). We did not observe HDBR-induced deterioration in performance of cognitive measures, neither relative to a pre-HDBR baseline, nor compared to the performance time course of normative control subjects. This is in line with previous HDBR studies that also found no significant detrimental effects of HDBR (Seaton et al., 2009) or even performance improvement over the course of HDBR on measures of psychomotor performance (Pavy-Le Traon et al., 1994), encoding (DeRoshia and Greenleaf, 1993), and 2D mental rotation (Liao et al., 2014).

The cognitive deterioration and cognitive improvements in our HDBR subjects, pre-HDBR, during-HDBR, or post-HDBR, were no longer significant after correction for multiple comparisons. Likewise, we did not observe any significant improvements in cognitive functioning in normative control subjects tested in the supine position after their second assessment after adjustment for multiple testing. Thus, no further practice effects were observed in normative subjects from their second assessment onwards. The fact that we did not find interaction effects between changes over time in HDBR subjects during HDBR and in normative control subjects over a similar time interval on any of our cognitive outcome measures implies that there are no effects of HDBR on the cognitive outcome measures under study, at least within the context of our study. However, the small sample size of our study may have resulted in type II errors. Therefore, HBDR studies with a larger sample size are warranted to verify the potential meaningfulness of the cognitive changes we observed that did not survive multiple comparisons correction.

In contrast with the lack of association between HDBR and psychomotor speed that we report, a previous 5 weeks no-tilt bed rest study in eight airmen reported detrimental effects of bed rest on a simple reaction time test that involves lever pressing and a measure of hand steadiness that requires putting a stylus in various sized holes while trying to avoid touching the edges of the holes (Ryback et al., 1971). However, exercise did not mitigate the effects of bed rest on psychomotor performance. A potential explanation for the discrepancies between this study and the current study could be that the psychomotor measures used in the study by Ryback et al. (1971) are more sensitive to the effects of bed rest. A few other studies have observed significant adverse effects of HDBR on cognitive functioning. For example, Seaton et al. (2007) reported that general cognitive performance in HDBR subjects was worse than in control subjects. However, these conclusions were based on descriptive statistics and no formal tests were conducted to test these differences. Another HDBR study by Lipnicki et al. (2009b) showed that 50 days of HDBR did not result in worse executive functioning, but rather that HDBR prevented improvements. In contrast, our comparison of changes in cognitive performance between HDBR subjects and normative control subjects found no evidence for an increase in performance being prevented by HDBR. The design used by Lipnicki et al. neatly prevents practice effects, but requires more subjects as it is a between subjects design (pre and post metrics were compared between differing subjects). Another study investigating effects of HDBR on executive functioning measured with a flanker task reported slower responses during HDBR compared to pre-HDBR (Liu et al., 2012). A potential explanation for the absence of significant deterioration of cognitive functioning in our study is that HDBR affects some but not all cognitive domains.

Our results indicate that long-duration HDBR does not cause cognitive deterioration in the domains under study. However, our small sample size, practice effects, and subjects’ cognitive reserve could have prevented us from finding significant effects of HDBR. Some of our outcome measures showed non-significant deterioration after 7 days of HDBR. Whether any detrimental effect of HDBR would be significant in larger samples should be a topic of future research, although based on our data, large effects are not to be expected. Practice effects refer to improved performance that can result from familiarity with the test procedure and material (Bartels et al., 2010). Using alternative versions of cognitive tests in future studies might partially prevent practice effects. Neural compensation dictates that individual differences in efficiency, capacity or flexibility allow better coping with disruption of adverse cognitive events (Stern, 2009). Considering their relatively young age, the subjects under study might have had sufficient cognitive reserve to withstand the cognitive effects of HDBR. Functional MRI (fMRI) studies could help answer the question if cognitive performance over the course of HDBR is associated with functional brain changes (Qin et al., 2003; Chein and Schneider, 2005).

### Sensorimotor Performance

We found that HDBR resulted in deterioration of FMT and standing balance performance. The deterioration appeared larger in HDBR control subjects than in exercise subjects and recovery of FMT post-HDBR was significantly more pronounced in the exercise subjects. The detrimental changes in FMT performance and balance remained significant after Bonferroni correction for multiple comparisons, although the mitigating effects of exercise did not survive. In comparison with normative control subjects, the HDBR-related performance drop in FMT of HDBR control subjects, but not that of HDBR exercise subjects, was significant. This supports the idea that exercise is an effective countermeasure for the HDBR effects on FMT. However, the aerobic and resistance exercise did not fully mitigate the effects of HDBR on gait and balance. Additional interventions such as supine balance training could further help HDBR effects on balance performance.

Our observations corroborate with previous HDBR studies examining sensorimotor performance that have shown similar detrimental effects of HDBR on FMT and standing balance performance after long-duration HDBR. A 60 days HDBR study conducted in our lab showed that HDBR leads to more time needed to complete an obstacle course that was similar to the one we used in our current study (Reschke et al., 2009). Interestingly, subjects in our previous study who received daily foot massages (and thus receive tactile and pressure input throughout HDBR) were less affected by HDBR in their performance on the obstacle course than those subjects that did not receive foot massages. Potentially, exercise combined with foot sole massage, and other interventions including balance training (Welch et al., 1993) could synergistically mitigate the detrimental effects of HDBR on gait and balance. A 30-day HDBR study showed that HDBR led to decreased step length, decreased walking velocity, and balance instability (Dupui et al., 1992). Finally, a 5 day HDBR study did not find evidence for detrimental effects of HDBR on gait, although it did show that post-HDBR, head movement resulted in postural instability and there was in increased incidence of falls (Mulder et al., 2014). The latter could be caused by posture control deficits similar to what we observed, whereas the absence of changes in gait might be due to the short duration of the HDBR. Using a crossover design, the authors showed that these effects of HDBR were successfully countered by either 25 min of daily upright standing or 25 min of locomotion-like exercise. This shows that loading can mitigate the effects of HDBR on gait, although it remains to be determined if solely loading is enough to also mitigate effects of long-duration HDBR.

HDBR-induced deterioration in motor performance has been ascribed to muscle unloading (Dilani Mendis et al., 2009), but may also be partially explained by CNS changes. The cephalad fluid shift in HDBR could lead to an increased intracranial pressure that in turn dysregulates functioning of brain regions, including those that are important for neuromotor control (Liao et al., 2012; Zhou et al., 2014).

The effects of HDBR on FMT and posture control are in line with the effects of spaceflight on these sensorimotor measures. Spaceflight can result in adaptive modification of walking strategies (Bloomberg and Mulavara, 2003; Mulavara et al., 2010). A study conducted in our lab showed that after 6 months of spaceflight, astronauts (*n* = 15) performed worse on the FMT and SOT-5 tests compared to pre-flight assessments (Cohen et al., 2012). Our results indicate that aerobic and resistance exercise is an effective, though not a fully curative, countermeasure for HDBR-related FMT deterioration, and therefore implicate that it could also be a suited countermeasure for the effects of microgravity during and post-spaceflight. The effect of HDBR on standing balance performance was not mitigated by exercise. Targeted interventions such as balance training and motor variability training could improve prevention of HDBR induced FMT deterioration and might be successful in the prevention of HDBR induced postural instability.

Our study is not without limitations. The small sample size of both our HDBR and normative control groups may have prevented our ability to detect small but potentially meaningful detrimental cognitive effects of head down bed rest. In addition, we did not collect information on emotional well-being and data on physical and muscle function have not yet been completely analyzed by the laboratory leading investigation of these metrics. Therefore, we could not adjust for these potential confounders in our analyses. Furthermore, our exercise protocol included both aerobic and resistance exercise. It is therefore not possible to pinpoint the mitigating effects of exercise on sensorimotor performance post-HDBR to either of the two. Finally, the differential interval and number of assessment time points between HDBR subjects and normative control subjects may have affected our outcomes. Studies with larger samples, with separate aerobic and resistance exercise groups, and which have the same number of assessments and equal length intervals for HDBR subjects and normative subjects are warranted.

The current study was conducted to investigate effects of a microgravity analog on cognitive and sensorimotor performance for which the results would translate to the effects of spaceflight on astronauts. However, long duration HDBR could also serve as model to investigate the combined effects of supine body orientation and inactivity that translate to temporarily or permanently bedridden individuals such as pregnant women, or to the large population of elderly residents of nursing homes. Indeed, a previous study in 680 non-disabled community-living persons aged 70 years and older reported that duration of bed rest was associated with decline in activities of daily living, mobility, physical activity and social activity (Gill et al., 2004). Exercise interventions could potentially also serve as a countermeasure for the bed rest related functional deterioration observed in this population.

## Conclusion

Seventy days of six-degree HDBR did not significantly adversely affect cognitive performance in our sample of 10 HDBR subjects. However, our results show that visual dependency during bed rest remained stable in HDBR control subjects whereas performance improved in HDBR exercise subjects. Furthermore, HDBR was associated with loss of FMT and standing balance performance, both of which almost fully recovered 12 days after the bed rest intervention had ended. Aerobic and resistance exercise partially mitigates the effects of HDBR on FMT and can speed up the recovery time course post-HDBR. The mechanisms underlying HDBR effects on sensorimotor performance are not yet fully understood and could include various factors such as muscle atrophy but also CNS changes. Neuroimaging HDBR studies could provide new insights into the potential plasticity of the CNS related to performance changes that result from HDBR. These insights would not only apply to astronauts, but could also translate to individuals who are temporarily or permanently bedridden, or individuals with reduced mobility.

## Funding

This work is supported by the National Space Biomedical Research Institute (NASA NCC 9-58, MA02701, and PF04101), grants from the National Aeronautics and Space Administration (NASA; NNX11AR02G) and NASA Flight Analogs Project, National Institutes of Health, and National Center for Advancing Translational Sciences, 1UL1RR029876-01.

## Conflict of Interest Statement

The authors declare that the research was conducted in the absence of any commercial or financial relationships that could be construed as a potential conflict of interest.

## References

[B1] Bartels C. Wegrzyn M. Wiedl A. Ackermann V. Ehrenreich H. (2010). Practice effects in healthy adults: a longitudinal study on frequent repetitive cognitive testing. BMC Neurosci. 11:118. 10.1186/1471-2202-11-118 20846444 PMC2955045

[B2] Bherer L. Erickson K. I. Liu-Ambrose T. (2013). A review of the effects of physical activity and exercise on cognitive and brain functions in older adults. J. Aging Res. 2013:657508. 10.1155/2013/657508 24102028 PMC3786463

[B3] Bloomberg J. J. Mulavara A. P. (2003). Changes in walking strategies after spaceflight. IEEE Eng. Med. Biol. Mag. 22, 58–62. 10.1109/memb.2003.1195697 12733460

[B4] Brady R. A. Peters B. T. Batson C. D. Ploutz-Snyder R. Mulavara A. P. Bloomberg J. J. (2012). Gait adaptability training is affected by visual dependency. Exp. Brain Res. 220, 1–9. 10.1007/s00221-012-3109-5 22585123

[B5] Calamia M. Markon K. Tranel D. (2012). Scoring higher the second time around: meta-analyses of practice effects in neuropsychological assessment. Clin. Neuropsychol. 26, 543–570. 10.1080/13854046.2012.680913 22540222

[B6] Caprihan A. Sanders J. A. Cheng H. A. Loeppky J. A. (1999). Effect of head-down tilt on brain water distribution. Eur. J. Appl. Physiol. Occup. Physiol. 79, 367–373. 10.1007/s004210050522 10090638

[B7] Chein J. M. Schneider W. (2005). Neuroimaging studies of practice-related change: fMRI and meta-analytic evidence of a domain-general control network for learning. Brain Res. Cogn. Brain Res. 25, 607–623. 10.1016/j.cogbrainres.2005.08.013 16242923

[B8] Cohen H. S. Kimball K. T. Mulavara A. P. Bloomberg J. J. Paloski W. H. (2012). Posturography and locomotor tests of dynamic balance after long-duration spaceflight. J. Vestib. Res. 22, 191–196. 10.3233/VES-2012-0456 23142833 PMC8080311

[B9] Convertino V. A. Bloomfield S. A. Greenleaf J. E. (1997). An overview of the issues: physiological effects of bed rest and restricted physical activity. Med. Sci. Sports Exerc. 29, 187–190. 10.1097/00005768-199702000-00004 9044221

[B10] de Boer M. R. Waterlander W. E. Kuijper L. Steenhuis I. Twisk J. (2015). Testing for baseline differences in randomized controlled trials: an unhealthy research behavior that is hard to eradicate. Int. J. Behav. Nutr. Phys. Act. 12:4. 10.1186/s12966-015-0162-z 25616598 PMC4310023

[B11] DeRoshia C. W. Greenleaf J. E. (1993). Performance and mood-state parameters during 30-day 6 degrees head-down bed rest with exercise training. Aviat. Space Environ. Med. 64, 522–527. 8338499

[B12] Dilani Mendis M. Hides J. A. Wilson S. J. Grimaldi A. Belavý D. L. Stanton W.. (2009). Effect of prolonged bed rest on the anterior hip muscles. Gait Posture 30, 533–537. 10.1016/j.gaitpost.2009.08.002 19726188

[B13] Dupui P. Montoya R. Costes-Salon M. C. Séverac A. Güell A. (1992). Balance and gait analysis after 30 days -6 degrees bed rest: influence of lower-body negative-pressure sessions. Aviat. Space Environ. Med. 63, 1004–1010. 1445150

[B14] Ekstrome R. French J. Harman H. (1976). Manual for kit of factor referenced cognitivetests. Princeton, New Jersey: Educational Testing Service.

[B15] Gill T. M. Allore H. Guo Z. (2004). The deleterious effects of bed rest among community-living older persons. J. Gerontol. A Biol. Sci. Med. Sci. 59, 755–761. 10.1093/gerona/59.7.m755 15304541

[B16] Holstein G. R. Kukielka E. Martinelli G. P. (1999). Anatomical observations of the rat cerebellar nodulus after 24 hr of spaceflight. J. Gravit. Physiol. 6, P47–P50. 11543023

[B17] Hutchison A. (2001). NASA Ames Seeks Volunteers for Month-Long Bed Rest Study. Available online at: http://www.nasa.gov/centers/ames/news/releases/2001/01_76AR.html. (accessed January 1, 2015).

[B18] Isableu B. Gueguen M. Fourré B. Giraudet G. Amorim M. A. (2008). Assessment of visual field dependence: comparison between the mechanical 3D rod-and-frame test developed by Oltman in 1968 with a 2D computer-based version. J. Vestib. Res. 18, 239–247. 19542598

[B19] Isableu B. Ohlmann T. Crémieux J. Amblard B. (1998). How dynamic visual field dependence-independence interacts with the visual contribution to postural control. Hum. Mov. Sci. 17, 367–391. 10.1016/s0167-9457(98)00005-0

[B20] Koppelmans V. Erdeniz B. De Dios Y. E. Wood S. J. Reuter-Lorenz P. A. Kofman I.. (2013). Study protocol to examine the effects of spaceflight and a spaceflight analog on neurocognitive performance: extent, longevity and neural bases. BMC Neurol. 13:205. 10.1186/1471-2377-13-205 24350728 PMC3878338

[B21] Lezak M. D. Howieson D. B. Loring D. W. (2004). Neuropsychological Assessment. New York: Oxford University Press.

[B22] Liao Y. Miao D. Huan Y. Yin H. Xi Y. Liu X. (2014). Altered regional homogeneity with short-term simulated microgravity and its relationship with changed performance in mental transformation. PLoS One 8:e64931. 10.1371/journal.pone.0064931 23755162 PMC3670926

[B23] Liao Y. Zhang J. Huang Z. Xi Y. Zhang Q. Zhu T.. (2012). Altered baseline brain activity with 72 h of simulated microgravity–initial evidence from resting-state fMRI. PLoS One 7:e52558. 10.1371/journal.pone.0052558 23285086 PMC3528642

[B24] Lipnicki D. M. Gunga H. C. (2009). Physical inactivity and cognitive functioning: results from bed rest studies. Eur. J. Appl. Physiol. 105, 27–35. 10.1007/s00421-008-0869-5 18797919

[B25] Lipnicki D. M. Gunga H. C. Belavý D. L. Felsenberg D. (2009a). Bed rest and cognition: effects on executive functioning and reaction time. Aviat. Space Environ. Med. 80, 1018–1024. 10.3357/asem.2581.2009 20027848

[B26] Lipnicki D. M. Gunga H. C. Belavy D. L. Felsenberg D. (2009b). Decision making after 50 days of simulated weightlessness. Brain Res. 1280, 84–89. 10.1016/j.brainres.2009.05.022 19447099

[B27] Liu Q. Zhou R. Chen S. Tan C. (2012). Effects of head-down bed rest on the executive functions and emotional response. PLoS One 7:e52160. 10.1371/journal.pone.0052160 23284916 PMC3524097

[B28] McGinnis P. M. (2013). Biomechanics of Sport and Exercise. Champaign, IL: Human Kinetics Publishers.

[B29] Moher D. Hopewell S. Schulz K. F. Montori V. Gøtzsche P. C. Devereaux P. J.. (2010). CONSORT 2010 explanation and elaboration: updated guidelines for reporting parallel group randomised trials. J. Clin. Epidemiol. 63, e1–e37. 10.1016/j.jclinepi.2010.03.004 20346624

[B30] Muir J. Judex S. Qin Y. X. Rubin C. (2011). Postural instability caused by extended bed rest is alleviated by brief daily exposure to low magnitude mechanical signals. Gait Posture 33, 429–435. 10.1016/j.gaitpost.2010.12.019 21273076 PMC3050431

[B31] Mulavara A. P. Feiveson A. H. Fiedler J. Cohen H. Peters B. T. Miller C.. (2010). Locomotor function after long-duration space flight: effects and motor learning during recovery. Exp. Brain Res. 202, 649–659. 10.1007/s00221-010-2171-0 20135100

[B32] Mulder E. Linnarsson D. Paloski W. H. Rittweger J. Wuyts F. L. Zange J.. (2014). Effects of five days of bed rest with and without exercise countermeasure on postural stability and gait. J. Musculoskelet Neuronal Interact. 14, 359–366. 25198232

[B34] Neurocom (2012). Sensory Organization Test (SOT). Available online at: http://resourcesonbalance.com/neurocom/protocols/sensoryimpairment/SOT.aspx.

[B33] Parihar V. K. Allen B. Tran K. K. Macaraeg T. G. Chu E. M. Kwok S. F. (2015). What happens to your brain on the way to Mars. Cogn. Neurosci. 4:e1400256 10.1126/sciadv.1400256 PMC4500198 26180843

[B35] Pavy-Le Traon A. Rous De Feneyrols A. Cornac A. Abdeseelam R. N’uygen D. Lazerges M.. (1994). Psychomotor performance during a 28 day head-down tilt with and without lower body negative pressure. Acta Astronaut. 32, 319–330. 10.1016/0094-5765(94)90083-3 11540777

[B36] Ploutz-Snyder L. L. Downs M. Ryder J. Hackney K. Scott J. Buxton R.. (2014). Integrated resistance and aerobic exercise protects fitness during bed rest. Med. Sci. Sports Exerc. 46, 358–368. 10.1249/mss.0b013e3182a62f85 24441215

[B37] Qin Y. Sohn M. H. Anderson J. R. Stenger V. A. Fissell K. Goode A.. (2003). Predicting the practice effects on the blood oxygenation level-dependent (BOLD) function of fMRI in a symbolic manipulation task. Proc. Natl. Acad. Sci. U S A 100, 4951–4956. 10.1073/pnas.0431053100 12672965 PMC153661

[B38] Rao L. L. Zhou Y. Liang Z. Y. Rao H. Zheng R. Sun Y.. (2014). Decreasing ventromedial prefrontal cortex deactivation in risky decision making after simulated microgravity: effects of -6 degrees head-down tilt bed rest. Front. Behav. Neurosci. 8:187. 10.3389/fnbeh.2014.00187 24904338 PMC4034329

[B39] Reschke M. F. Bloomberg J. J. Paloski W. H. Mulavara A. P. Feiveson A. H. Harm D. L. (2009). Postural reflexes, balance control and functional mobility with long-duration head-down bed rest. Aviat. Space Environ. Med. 80, A45–A54. 10.3357/asem.br06.2009 19476169

[B40] Roberts D. R. Ramsey D. Johnson K. Kola J. Ricci R. Hicks C.. (2010). Cerebral cortex plasticity after 90 days of bed rest: data from TMS and fMRI. Aviat. Space Environ. Med. 81, 30–40. 10.3357/asem.2532.2009 20058735 PMC2861654

[B41] Ryback R. S. Trimble R. W. Lewis O. F. Jennings C. L. (1971). Psychobiologic effects of prolonged weightlessness (bed rest) in young healthy volunteers. Aerosp. Med. 42, 408–415. 4343907

[B42] Seaton K. A. Slack K. J. Sipes W. Bowie K. (2007). Artificial gravity as a multi-system countermeasure: effects on cognitive function. J. Gravit Physiol. 14, P27–P30. 18372688

[B43] Seaton K. A. Slack K. J. Sipes W. A. Bowie K. E. (2009). Cognitive functioning in long-duration head-down bed rest. Aviat. Space Environ. Med. 80, A62–A65. 10.3357/asem.br09.2009 19476171

[B44] Shepard S. Metzler D. (1988). Mental rotation: effects of dimensionality of objects and type of task. J. Exp. Psychol. Hum. Percept. Perform. 14, 3–11. 10.1037/0096-1523.14.1.3 2964504

[B45] Stern Y. (2009). Cognitive reserve. Neuropsychologia 47, 2015–2028. 10.1016/j.neuropsychologia.2009.03.004 19467352 PMC2739591

[B46] Strangman G. E. Sipes W. Beven G. (2014). Human cognitive performance in spaceflight and analogue environments. Aviat. Space Environ. Med. 85, 1033–1048. 10.3357/ASEM.3961.2014 25245904

[B47] Tiffin J. Asher E. J. (1948). The purdue pegboard; norms and studies of reliability and validity. J. Appl. Psychol. 32, 234–247. 10.1037/h0061266 18867059

[B48] Van Dongen S. Molenberghs G. Matthysen E. (1999). The statistical analysis of fluctuating assymetry: REML estimation of a mixed regression model. J. Evol. Biol. 12, 94–102. 10.1046/j.1420-9101.1999.00012.x

[B49] Welch R. B. Bridgeman B. Anand S. Browman K. E. (1993). Alternating prism exposure causes dual adaptation and generalization to a novel displacement. Percept. Psychophys. 54, 195–204. 10.3758/bf03211756 8361835

[B50] Wood S. J. Reschke M. F. Owen Black F. (2012). Continuous equilibrium scores: factoring in the time before a fall. Gait Posture 36, 487–489. 10.1016/j.gaitpost.2012.04.014 22640866

[B51] Young L. R. Oman C. M. Watt D. G. Money K. E. Lichtenberg B. K. (1984). Spatial orientation in weightlessness and readaptation to earth’s gravity. Science 225, 205–208. 10.1126/science.6610215 6610215

[B52] Young L. R. Oman C. M. Watt D. G. Money K. E. Lichtenberg B. K. Kenyon R. V.. (1986). M.I.T./Canadian vestibular experiments on the Spacelab-1 mission: 1. Sensory adaptation to weightlessness and readaptation to one-g: an overview. Exp. Brain Res. 64, 291–298. 10.1007/bf00237746 3492384

[B53] Zhou Y. Wang Y. Rao L.-L. Liang Z.-Y. Chen X.-P. Zheng D.. (2014). Disrupted resting-state functional architecture of the brain after 45-day simulated microgravity. Front. Behav. Neurosci. 8:200. 10.3389/fnbeh.2014.00200 24926242 PMC4046318

